# The genome sequence of the tree-moss, *Climacium dendroides *(Hedw.) F.Weber & D.Mohr (Climaciaceae)

**DOI:** 10.12688/wellcomeopenres.22450.1

**Published:** 2024-06-13

**Authors:** David Bell, David G. Long, Royal Botanic Garden Edinburgh Genome Acquisition Lab, Plant Genome Sizing collective, Darwin Tree of Life Barcoding collective, Wellcome Sanger Institute Tree of Life Management, Samples and Laboratory team, Wellcome Sanger Institute Scientific Operations: Sequencing Operations, Wellcome Sanger Institute Tree of Life Core Informatics team, Tree of Life Core Informatics collective, Darwin Tree of Life Consortium

**Affiliations:** 1 Royal Botanic Garden Edinburgh, Edinburgh, Scotland, UK

**Keywords:** Climacium dendroides, tree-moss, genome sequence, chromosomal, Leucodontales

## Abstract

We present a genome assembly from an individual *Climacium dendroides* gametophyte (the tree-moss; Bryophyta; Bryopsida; Leucodontales; Climaciaceae). The genome sequence is 413.1 megabases in span. Most of the assembly is scaffolded into 11 chromosomal pseudomolecules. The mitochondrial and plastid genome assemblies have lengths of 104.86 kilobases and 124.96 kilobases in length, respectively.

## Species taxonomy

Eukaryota; Viridiplantae; Streptophyta; Streptophytina; Embryophyta; Bryophyta; Bryophytina; Bryopsida; Bryidae; Hypnanae; Hypnales; Climaciaceae; *Climacium*, *Climacium dendroides* (Hedw.) F.Weber & D.Mohr (NCBI:txid70131).

## Background

*Climacium dendroides* (Hedw.) F.Weber & D.Mohr, also known as tree-moss, is a large pleurocarpous moss with a tree-like habit. Common in the north and west of Britain and Ireland, its native range extends across the northern hemisphere, with disjunct populations (likely introduced) in Australia and New Zealand ( Blockeel *et al.*, 2014). It is found in a range of habitats, but prefers damp, mildly basic ground where water levels fluctuate, such as damp grassland, dune-slacks and marshy ground.

*Climacium dendroides* is a dioicous species, producing male and female reproductive structures on separate plants. Although sex organs are commonly produced in damp habitats, most colonies are unisexual and capsules are therefore rarely produced in Britain and Ireland ( Blockeel *et al.*, 2014).

The assembled genome presented here is consistent with published chromosome counts ( Fritsch, 1991) and genome size estimates ( Voglmayr, 2000), although Bainard *et al.* ( Bainard *et al.*, 2020) report a genome size estimate of 983 Mb for a Canadian sample, suggesting a possible polyploid. We anticipate this high-quality reference genome will be a valuable genomic resource for a range of future studies.

## Genome sequence report

**Figure 1.**
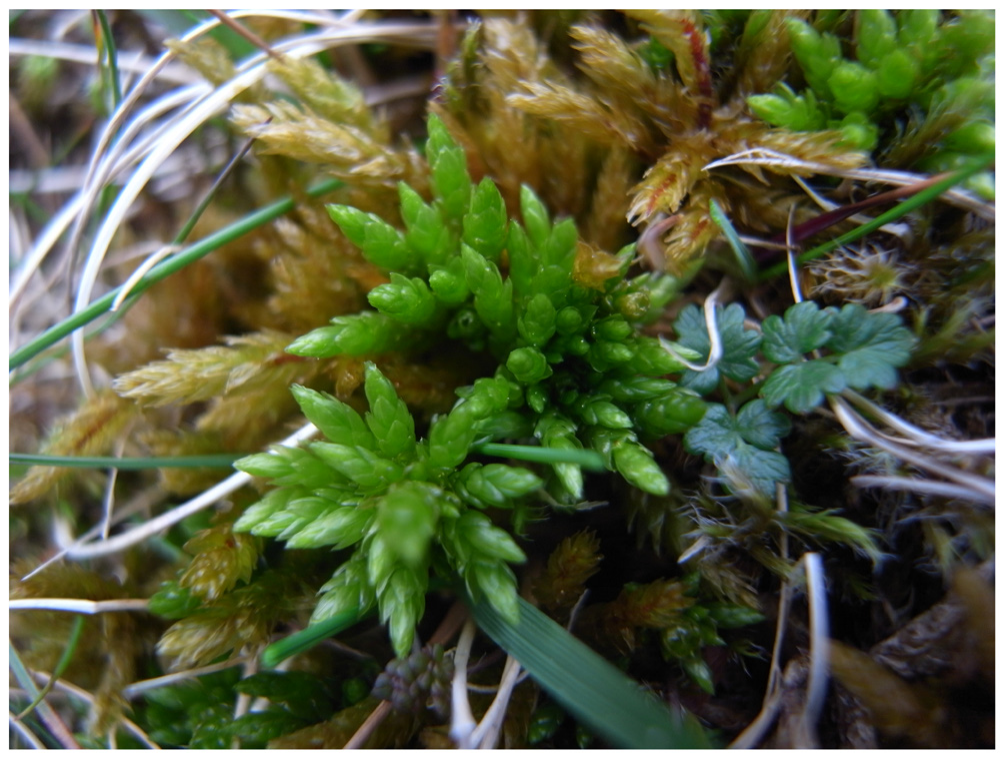
Photograph of the *Climacium dendroides* population from which samples used for genome sequencing were taken.

The genome was sequenced from one specimen of *Climacium dendroides* ( Figure 1) collected from Breadalbane Mountains, Perthshire, Scotland, UK (56.54, –4.23). Using flow cytometry, the genome size (1C-value) was estimated to be 0.52 pg, equivalent to 510 Mb. A total of 38-fold coverage in Pacific Biosciences single-molecule HiFi long reads was generated. Primary assembly contigs were scaffolded with chromosome conformation Hi-C data. Manual assembly curation corrected 9 missing joins, increasing the scaffold number by 2.33%.

**Figure 2.**
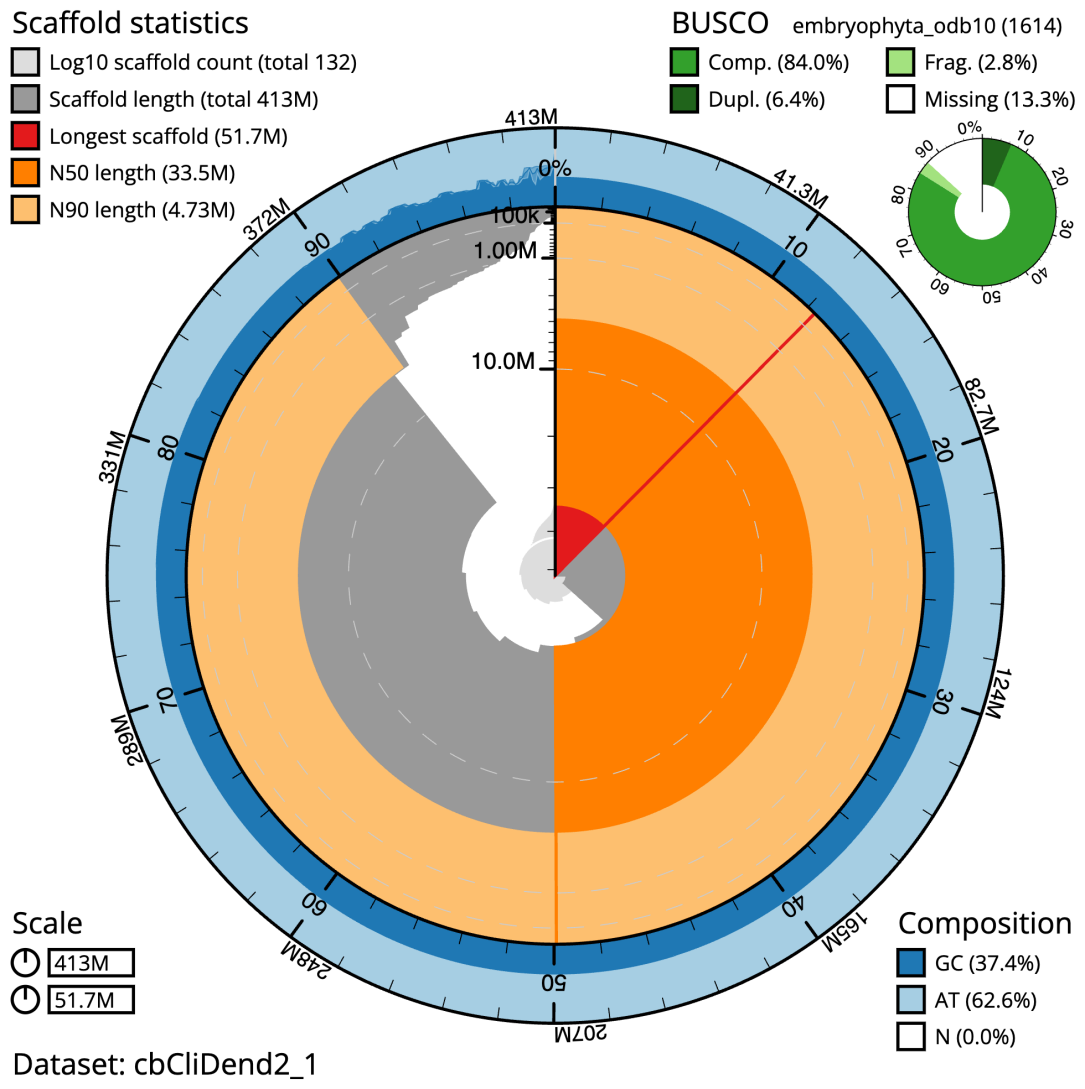
Genome assembly of *Climacium dendroides*, cbCliDend2.1: metrics. The BlobToolKit Snailplot shows N50 metrics and BUSCO gene completeness. The main plot is divided into 1,000 size-ordered bins around the circumference with each bin representing 0.1% of the 413,289,925 bp assembly. The distribution of scaffold lengths is shown in dark grey with the plot radius scaled to the longest scaffold present in the assembly (51,654,917 bp, shown in red). Orange and pale-orange arcs show the N50 and N90 scaffold lengths (33,543,809 and 4,731,204 bp), respectively. The pale grey spiral shows the cumulative scaffold count on a log scale with white scale lines showing successive orders of magnitude. The blue and pale-blue area around the outside of the plot shows the distribution of GC, AT and N percentages in the same bins as the inner plot. A summary of complete, fragmented, duplicated and missing BUSCO genes in the embryophyta_odb10 set is shown in the top right. An interactive version of this figure is available at https://blobtoolkit.genomehubs.org/view/cbCliDend2_1/dataset/cbCliDend2_1/snail.

**Figure 3.**
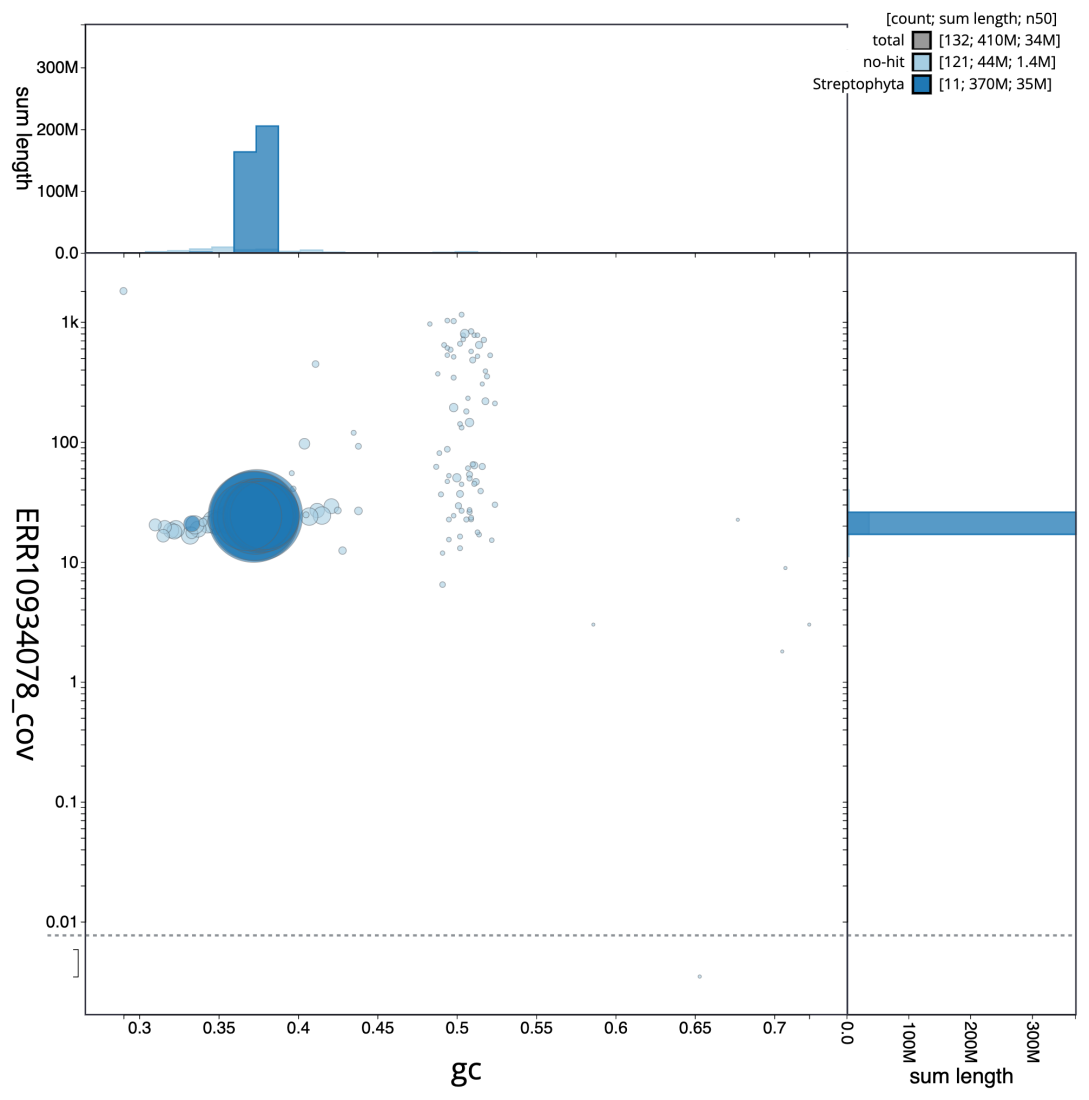
Genome assembly of *Climacium dendroides*, cbCliDend2.1: BlobToolKit GC-coverage plot. Scaffolds are coloured by phylum. Circles are sized in proportion to scaffold length. Histograms show the distribution of scaffold length sum along each axis. An interactive version of this figure is available at https://blobtoolkit.genomehubs.org/view/cbCliDend2_1/dataset/cbCliDend2_1/blob.

**Figure 4.**
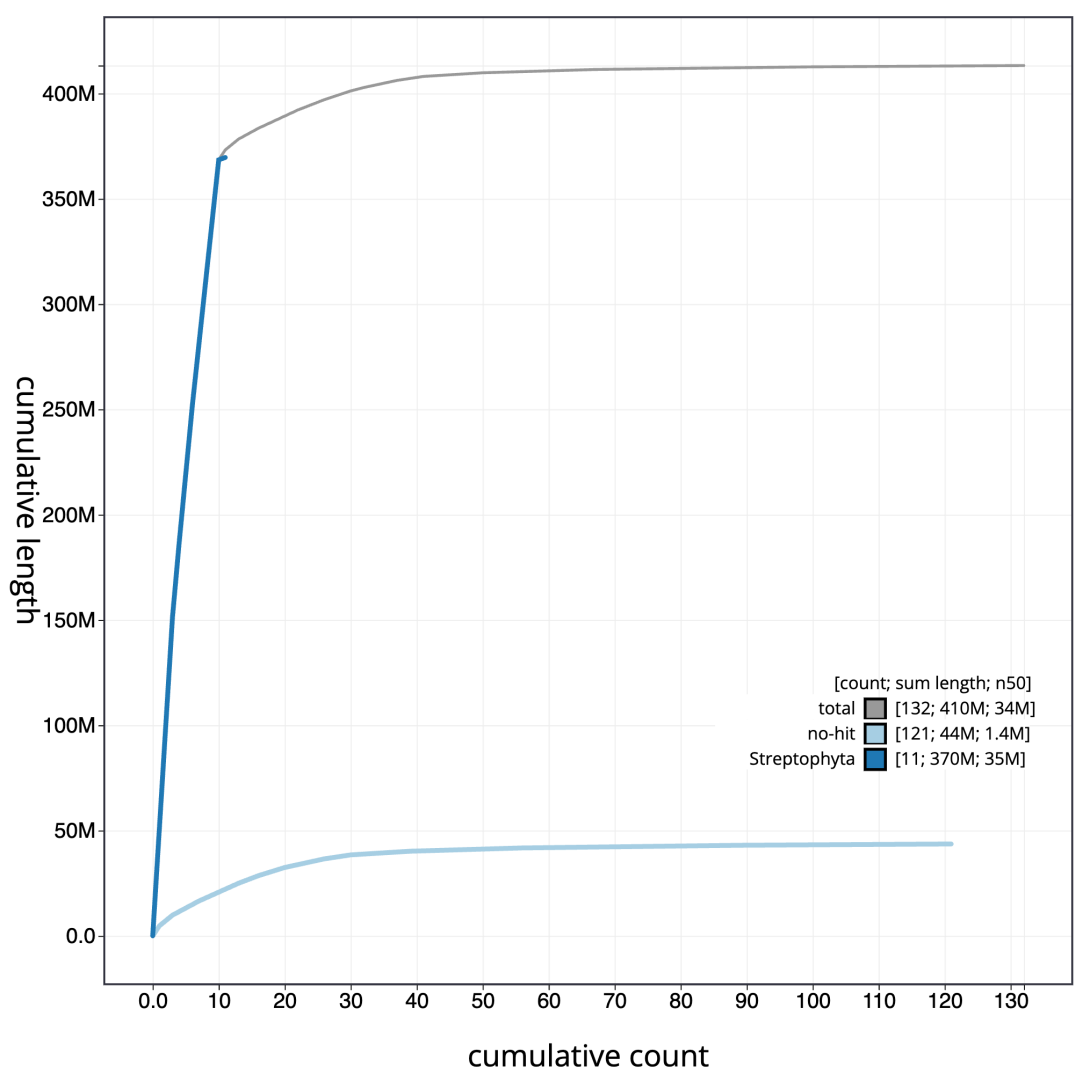
Genome assembly of *Climacium dendroides*, cbCliDend2.1: BlobToolKit cumulative sequence plot. The grey line shows cumulative length for all scaffolds. Coloured lines show cumulative lengths of scaffolds assigned to each phylum using the buscogenes taxrule. An interactive version of this figure is available at https://blobtoolkit.genomehubs.org/view/cbCliDend2_1/dataset/cbCliDend2_1/cumulative.

**Figure 5.**
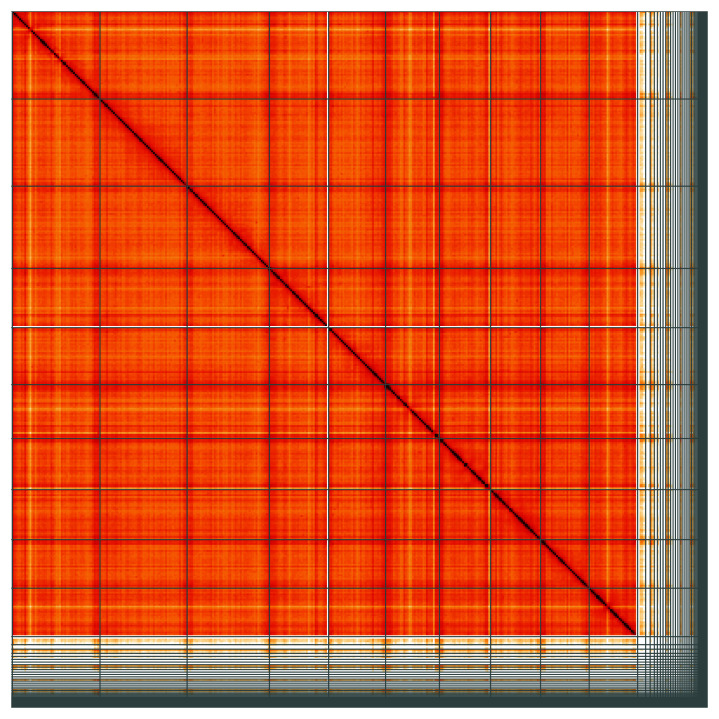
Genome assembly of *Climacium dendroides*, cbCliDend2.1: Hi-C contact map of the cbCliDend2.1 assembly, visualised using HiGlass. Chromosomes are shown in order of size from left to right and top to bottom. An interactive version of this figure may be viewed at https://genome-note-higlass.tol.sanger.ac.uk/l/?d=EIC-OixDRXS2EoTxupeZJg.

The final assembly has a total length of 413.1 Mb in 130 sequence scaffolds with a scaffold N50 of 33.5 Mb ( Table 1). The snail plot in Figure 2 provides a summary of the assembly statistics, while the distribution of assembly scaffolds on GC proportion and coverage is shown in Figure 3. The cumulative assembly plot in Figure 4 shows curves for subsets of scaffolds assigned to different phyla. Most (98.65%) of the assembly sequence was assigned to 11 chromosomal-level scaffolds. Chromosome-scale scaffolds confirmed by the Hi-C data are named in order of size ( Figure 5; Table 2). Chromosome 4 is likely the U or V sex chromosome. The PacBio data (used for *de novo* assembly) and Hi-C data (used for scaffolding) data are from different samples. The Hi-C data do not map to chromosome 4,which is not scaffolded due to lack of signal ( Figure 5), indicating that this chromosome is not present in the Hi-C sample. Sex is unknown for either sample. The mitochondrial and plastid genomes were also assembled and can be found as contigs within the multifasta file of the genome submission.

**Table 1.** Genome data for *Climacium dendroides*, cbCliDend2.1.

Project accession data		
Assembly identifier	cbCliDend2.1	
Species	*Climacium dendroides*	
Specimen	cbCliDend2	
NCBI taxonomy ID	70131	
BioProject	PRJEB60208	
BioSample ID	SAMEA10332383	
Isolate information	cbCliDend2 (unknown sex): gametophyte shoot (DNA and RNA sequencing) cbCliDend1 (unknown sex): gametophyte shoot (Hi-C sequencing)	
Assembly metrics *		*Benchmark*
Consensus quality (QV)	67.4	*≥ 50*
*k*-mer completeness	100.0%	*≥ 95%*
BUSCO **	C:84.0%[S:77.5%,D:6.4%],F:2.8%,M:13.3%,n:1,614	*C ≥ 95%*
Percentage of assembly mapped to chromosomes	98.65%	*≥ 95%*
Sex chromosomes	None	*localised homologous pairs*
Organelles	Mitochondrial genome: 104.86 kb Plastid genome: 124.96 kb	*complete single alleles*
Raw data accessions		
PacificBiosciences SEQUEL II	ERR10934077, ERR10934078	
Hi-C Illumina	ERR10936421	
PolyA RNA-Seq Illumina	ERR11242531	
Genome assembly		
Assembly accession	GCA_950106755.1	
Span (Mb)	413.1	
Number of contigs	204	
Contig N50 length (Mb)	6.6	
Number of scaffolds	130	
Scaffold N50 length (Mb)	33.5	
Longest scaffold (Mb)	51.65	

* Assembly metric benchmarks are adapted from column VGP-2020 of “Table 1: Proposed standards and metrics for defining genome assembly quality” from ( Rhie *et al.*, 2021). ** BUSCO scores based on the embryophyta_odb10 BUSCO set using version 5.3.2. C = complete [S = single copy, D = duplicated], F = fragmented, M = missing, n = number of orthologues in comparison. A full set of BUSCO scores is available at https://blobtoolkit.genomehubs.org/view/cbCliDend2_1/dataset/cbCliDend2_1/busco.

**Table 2.** Chromosomal pseudomolecules in the genome assembly of *Climacium dendroides*, cbCliDend2.

INSDC accession	Chromosome	Length (Mb)	GC%
OX467082.1	1	51.65	37.5
OX467083.1	2	51.35	37.0
OX467084.1	3	48.51	37.0
OX467085.1	4	4.73	36.0
OX467086.1	5	34.89	37.5
OX467087.1	6	33.54	37.5
OX467088.1	7	31.75	38.0
OX467089.1	8	30.15	37.5
OX467090.1	9	29.46	37.5
OX467091.1	10	28.63	38.0
OX467092.1	11	28.55	37.0
OX467093.1	MT	0.1	41.0
OX467094.1	Pltd	0.12	29.0

The estimated Quality Value (QV) of the final assembly is 67.4 with *k*-mer completeness of 100.0%, and the assembly has a BUSCO v5.3.2 completeness of 84.0% (single = 77.5%, duplicated = 6.4%), using the embryophyta_odb10 reference set ( *n* = 1,614).

Metadata for specimens, barcode results, spectra estimates, sequencing runs, contaminants and pre-curation assembly statistics are given at https://links.tol.sanger.ac.uk/species/70131.

## Methods

### Sample acquisition, genome size estimation and nucleic acid extraction

*Climacium dendroides* specimens were collected from Breadalbane Mountains, Mid Perthshire, Scotland, UK (latitude 56.54, longitude –4.23) on 2021-06-14. The living plants were wild-collected in tubs for processing in the lab. The plant used for DNA and RNA sequencing had specimen ID EDTOL01818 (ToLID cbCliDend2). The specimen used for Hi-C sequencing had specimen ID EDTOL01817 (ToLID cbCliDend1). The specimens were collected and identified by David Long and David Bell (Royal Botanic Garden Edinburgh), and were preserved by flash-freezing in liquid nitrogen. The herbarium specimen associated with the sequenced plants is kept at the Royal Botanic Garden Edinburgh (E) ( https://data.rbge.org.uk/herb/E01152115).

The genome size was estimated by flow cytometry using the fluorochrome propidium iodide and following the ‘one-step’ method as outlined in Pellicer *et al.* (2021). For this species, CyStain™ PI OxProtect Staining Buffer (cat. No. 05-5027; Sysmex UK Ltd.) was used for isolation of nuclei, and the internal calibration standard was *Oryza sativa* ‘IR36’ with an assumed 1C-value of 493.89 Mb ( Obermayer *et al.*, 2002).

The workflow for high molecular weight (HMW) DNA extraction at the Wellcome Sanger Institute (WSI) includes a sequence of core procedures: sample preparation; sample homogenisation, DNA extraction, fragmentation, and clean-up. In sample preparation, the cbCliDend2 sample was weighed and dissected on dry ice ( Jay *et al.*, 2023). For sample homogenisation, shoots were cryogenically disrupted using the Covaris cryoPREP ^®^ Automated Dry Pulverizer ( Narváez-Gómez *et al.*, 2023).

HMW DNA was extracted using the Automated Plant MagAttract v2 protocol ( Todorovic *et al.*, 2023a). HMW DNA was sheared into an average fragment size of 12–20 kb in a Megaruptor 3 system with speed setting 30 ( Todorovic *et al.*, 2023b). Sheared DNA was purified by automated solid-phase reversible immobilisation ( Oatley *et al.*, 2023): in brief, the method employs a 1.8X ratio of AMPure PB beads to sample to eliminate shorter fragments and concentrate the DNA. The concentration of the sheared and purified DNA was assessed using a Nanodrop spectrophotometer and Qubit Fluorometer and Qubit dsDNA High Sensitivity Assay kit. Fragment size distribution was evaluated by running the sample on the FemtoPulse system.

RNA was extracted from shoots of cbCliDend2 in the Tree of Life Laboratory at the WSI using the RNA Extraction: Automated MagMax™ *mir*Vana protocol ( do Amaral *et al.*, 2023). The RNA concentration was assessed using a Nanodrop spectrophotometer and a Qubit Fluorometer using the Qubit RNA Broad-Range Assay kit. Analysis of the integrity of the RNA was done using the Agilent RNA 6000 Pico Kit and Eukaryotic Total RNA assay.

Protocols developed by the WSI Tree of Life core laboratory are publicly available on protocols.io ( Denton *et al.*, 2023).

### Sequencing

Pacific Biosciences HiFi circular consensus DNA sequencing libraries were constructed according to the manufacturers’ instructions. Poly(A) RNA-Seq libraries were constructed using the NEB Ultra II RNA Library Prep kit. DNA and RNA sequencing was performed by the Scientific Operations core at the WSI on Pacific Biosciences SEQUEL II (HiFi) and Illumina NovaSeq 6000 (RNA-Seq) instruments. Hi-C data were also generated from shoot tissue of cbCliDend1 using the Arima2 kit and sequenced on the Illumina NovaSeq 6000 instrument.

### Genome assembly, curation and evaluation

Assembly was carried out with Hifiasm ( Cheng *et al.*, 2021). The assembly was scaffolded with Hi-C data ( Rao *et al.*, 2014), using YaHS ( Zhou *et al.*, 2023). The assembly was checked for contamination and corrected using the TreeVal pipeline ( Pointon *et al.*, 2023). Manual curation was performed using JBrowse2 ( Diesh *et al.*, 2023), HiGlass ( Kerpedjiev *et al.*, 2018) and PretextView ( Harry, 2022). The organelle genomes were assembled using MitoHiFi ( Uliano-Silva *et al.*, 2023) and OATK ( Zhou, 2023).

A Hi-C map for the final assembly was produced using bwa-mem2 ( Vasimuddin *et al.*, 2019) in the Cooler file format ( Abdennur & Mirny, 2020). To assess the assembly metrics, the *k*-mer completeness and QV consensus quality values were calculated in Merqury ( Rhie *et al.*, 2020). This work was done using Nextflow ( Di Tommaso *et al.*, 2017) DSL2 pipelines “sanger-tol/readmapping” ( Surana *et al.*, 2023a) and “sanger-tol/genomenote” ( Surana *et al.*, 2023b). The genome was analysed within the BlobToolKit environment ( Challis *et al.*, 2020) and BUSCO scores ( Manni *et al.*, 2021; Simão *et al.*, 2015) were calculated.

Table 3 contains a list of relevant software tool versions and sources.

**Table 3.** Software tools: versions and sources.

Software tool	Version	Source
BlobToolKit	4.1.7	https://github.com/blobtoolkit/blobtoolkit
BUSCO	5.3.2	https://gitlab.com/ezlab/busco
Hifiasm	0.16.1-r375	https://github.com/chhylp123/hifiasm
HiGlass	1.11.6	https://github.com/higlass/higlass
Merqury	MerquryFK	https://github.com/thegenemyers/MERQURY.FK
MitoHiFi	2	https://github.com/marcelauliano/MitoHiFi
OATK	0.1	https://github.com/c-zhou/oatk
PretextView	0.2	https://github.com/wtsi-hpag/PretextView
sanger-tol/genomenote	v1.0	https://github.com/sanger-tol/genomenote
sanger-tol/readmapping	1.1.0	https://github.com/sanger-tol/readmapping/tree/1.1.0
TreeVal	-	https://github.com/sanger-tol/treeval
YaHS	1.2a	https://github.com/c-zhou/yahs

### Wellcome Sanger Institute – Legal and Governance

The materials that have contributed to this genome note have been supplied by a Darwin Tree of Life Partner. The submission of materials by a Darwin Tree of Life Partner is subject to the **‘Darwin Tree of Life Project Sampling Code of Practice’**, which can be found in full on the Darwin Tree of Life website here. By agreeing with and signing up to the Sampling Code of Practice, the Darwin Tree of Life Partner agrees they will meet the legal and ethical requirements and standards set out within this document in respect of all samples acquired for, and supplied to, the Darwin Tree of Life Project. 

Further, the Wellcome Sanger Institute employs a process whereby due diligence is carried out proportionate to the nature of the materials themselves, and the circumstances under which they have been/are to be collected and provided for use. The purpose of this is to address and mitigate any potential legal and/or ethical implications of receipt and use of the materials as part of the research project, and to ensure that in doing so we align with best practice wherever possible. The overarching areas of consideration are:

• Ethical review of provenance and sourcing of the material

• Legality of collection, transfer and use (national and international) 

Each transfer of samples is further undertaken according to a Research Collaboration Agreement or Material Transfer Agreement entered into by the Darwin Tree of Life Partner, Genome Research Limited (operating as the Wellcome Sanger Institute), and in some circumstances other Darwin Tree of Life collaborators.

## Data Availability

European Nucleotide Archive: *Climacium dendroides (*tree-moss). Accession number PRJEB60208; https://identifiers.org/ena.embl/PRJEB60208 ( Wellcome Sanger Institute, 2023). The genome sequence is released openly for reuse. The *Climacium dendroides* genome sequencing initiative is part of the Darwin Tree of Life (DToL) project. All raw sequence data and the assembly have been deposited in INSDC databases. The genome will be annotated using available RNA-Seq data and presented through the Ensembl pipeline at the European Bioinformatics Institute. Raw data and assembly accession identifiers are reported in Table 1.

## Author information

Members of the Royal Botanic Garden Edinburgh Genome Acquisition Lab are listed here: https://doi.org/10.5281/zenodo.4786682.

Members of the Plant Genome Sizing collective are listed here: https://doi.org/10.5281/zenodo.7994306.

Members of the Darwin Tree of Life Barcoding collective are listed here: https://doi.org/10.5281/zenodo.4893703.

Members of the Wellcome Sanger Institute Tree of Life Management, Samples and Laboratory team are listed here: https://doi.org/10.5281/zenodo.10066175.

Members of Wellcome Sanger Institute Scientific Operations: Sequencing Operations are listed here: https://doi.org/10.5281/zenodo.10043364.

Members of the Wellcome Sanger Institute Tree of Life Core Informatics team are listed here: https://doi.org/10.5281/zenodo.10066637.

Members of the Tree of Life Core Informatics collective are listed here: https://doi.org/10.5281/zenodo.5013541.

Members of the Darwin Tree of Life Consortium are listed here: https://doi.org/10.5281/zenodo.4783558.
