# Supplementary material for: The genome sequence of the tree-moss, Climacium dendroides (Hedw.) F.Weber & D.Mohr (Climaciaceae)
Source: Wellcome Open Res. 2024 Jun 13;9:311. [Version 1] doi: 10.12688/wellcomeopenres.22450.1 (PMC11503002; doi:10.12688/wellcomeopenres.22450.1)
Supplement: Peer review history

# Reviewer report: Reviewer response for version 1

**DOI:** 10.21956/wellcomeopenres.24737.r107328

**Published:** 2024-11-04

**Authors:** Qilin Yang

**Affiliations:** 1 University of Chinese Academy of Sciences, Beijing, China

**Summary: **This data note presents the genome sequence assembly of *Climacium dendroides* (tree-moss), generated as part of the Darwin Tree of Life project. The authors report a high-quality chromosome-scale assembly spanning 413.1 Mb, with most (98.65%) of the sequence organized into 11 chromosomal pseudomolecules. The assembly includes complete mitochondrial and plastid genomes. The methods for sample collection, DNA/RNA extraction, sequencing, assembly and quality assessment are thoroughly documented.

**Minor Suggestions for Improvement:**

1. Consider including chromosome 4 in Figure 5 Hi-C contact map, even with low coverage, to illustrate its unique characteristics. 
2. Consider adding a phylogenetic tree showing evolutionary context of this species among mosses.
3. Characteristics of chromosome 4 and suspected sex chromosomes. The size of chromosome 4 is 4.73 Mb, which is significantly smaller than other chromosomes (the size of other chromosomes is between 28-51 Mb). The mapping signal of Hi-C data on this chromosome is missing, and the GC content is 36.0%, which is slightly lower than other chromosomes. (37.0-38.0%). It is recommended to conduct in-depth analysis later.
*Structural feature analysis:*
  - Chromosome size is significantly smaller than autosomes (approximately 1/6-1/10).
  - The GC content is low, indicating that there may be a special sequence composition.
  - May contain large amounts of repetitive sequences or regions of heterochromatin.
  Comparison of sex chromosome characteristics:
  - Sequence comparison with other known bryophyte sex chromosomes.
  - Analyze the evolutionary rate of homologous genes.
  - Detect the enrichment of repetitive sequences.

*Overall Assessment:* This is a well-documented genome sequence dataset that will be valuable for the research community. The methods are robust and thoroughly described, and the data is readily accessible. I recommend accepting this data note with minor revisions to address the suggestions above.

 The work meets the key criteria for scientific soundness and data availability for a genome sequence data note.

Are sufficient details of methods and materials provided to allow replication by others?

Yes

Is the rationale for creating the dataset(s) clearly described?

Yes

Are the datasets clearly presented in a useable and accessible format?

Yes

Are the protocols appropriate and is the work technically sound?

Yes

Reviewer Expertise:

Plant genomics and multi-omics.

I confirm that I have read this submission and believe that I have an appropriate level of expertise to confirm that it is of an acceptable scientific standard.

# Reviewer report: Reviewer response for version 1

**DOI:** 10.21956/wellcomeopenres.24737.r107327

**Published:** 2024-10-24

**Authors:** Facundo Romani

**Affiliations:** 1 Department of Plant Sciences, University of Cambridge, Cambridge, England, UK

The work being done by the Darwin Tree of Life project is of extreme importance for plant biologists across the globe. *Climacium dendroides *is a very ecologically important species. The methods are thoroughly described. To make the data even more usable, the annotation of the genome (.gff files) should be submitted with the Data Note. That will also provide extra analysis about the type of genes and homology with other related species. That kind of basic analysis should be part of the pipeline in future submissions. Another suggestion could be adding a phylogenetic tree of mosses or bryophyte to help the readers to appreciate the evolutionary position of this particular species.

Are sufficient details of methods and materials provided to allow replication by others?

Yes

Is the rationale for creating the dataset(s) clearly described?

Yes

Are the datasets clearly presented in a useable and accessible format?

Yes

Are the protocols appropriate and is the work technically sound?

Yes

Reviewer Expertise:

Plant molecular biology of bryophytes. Evo-devo.

I confirm that I have read this submission and believe that I have an appropriate level of expertise to confirm that it is of an acceptable scientific standard.

# Reviewer report: Reviewer response for version 1

**DOI:** 10.21956/wellcomeopenres.24737.r98594

**Published:** 2024-09-16

**Authors:** Hyoungseok Lee

**Affiliations:** 1 Korea Polar Research Institute, Incheon, South Korea

This manuscript presents a chromosome-scale assembly of *Climacium dendroides*. This work will undoubtedly serve as a valuable reference for studying evolution and environmental adaptations within the order Hypnales, to which most mosses belong. I suggest a few minor revisions for further clarification and improvement:

**# Chromosome 4 and sex chromosome issue**

 The discrepancy between HiFi and Hi-C sequencing samples raises an interesting possibility regarding chromosome 4. The authors' suggestion that chromosome 4 could be a sex chromosome due to insufficient Hi-C mapping is a reasonable hypothesis. To strengthen this claim, the following revisions are recommended:

 1. Include chromosome 4 in Figure 5, even with its low Hi-C support values. This visual representation would help readers understand the unique characteristics of this potential sex chromosome.

 2. Label all chromosomes (1 through 11) in Figure 5 for clarity and ease of reference.

**# Flow cytometry and genome size discrepancy**

 The explanation on difference between the predicted genome size (510 Mb) from flow cytometry and the assembly result (413 Mb) would greatly enhance the readers' understanding.

Are sufficient details of methods and materials provided to allow replication by others?

Yes

Is the rationale for creating the dataset(s) clearly described?

Yes

Are the datasets clearly presented in a useable and accessible format?

Yes

Are the protocols appropriate and is the work technically sound?

Yes

Reviewer Expertise:

bryophyte biology, genomics, ecology

I confirm that I have read this submission and believe that I have an appropriate level of expertise to confirm that it is of an acceptable scientific standard.
